# Systemic klotho is associated with *KLOTHO* variation and predicts intrinsic cortical connectivity in healthy human aging

**DOI:** 10.1007/s11682-016-9598-2

**Published:** 2016-10-06

**Authors:** Jennifer S. Yokoyama, Gabe Marx, Jesse A. Brown, Luke W. Bonham, Dan Wang, Giovanni Coppola, William W. Seeley, Howard J. Rosen, Bruce L. Miller, Joel H. Kramer, Dena B. Dubal

**Affiliations:** 1 Department of Neurology, University of California, San Francisco, 675 Nelson Rising Lane, San Francisco, CA 94158 USA; 2 Department of Neurology and Semel Institute for Neuroscience and Human Behavior, The David Geffen School of Medicine at University of California, Los Angeles, CA 90095 USA

**Keywords:** Aging, Cognition, Genetic variation, Longevity, Resilience, Klotho, Imaging, Connectivity, Frontal cortex

## Abstract

Cognitive decline is a major biomedical challenge as the global population ages. Elevated levels of the longevity factor klotho suppress aging, enhance cognition, and promote synaptic plasticity and neural resilience against aging and Alzheimer’s disease (AD)-related pathogenic proteins. Here, we examined the relationship between human genetic variants of *KLOTHO* and systemic klotho levels – and assessed neuroanatomic correlates of serum klotho in a cohort of healthy older adults. Serum klotho levels were increased with KL-VS heterozygosity, as anticipated. We report, for the first time, that serum klotho levels were paradoxically decreased with KL-VS homozygosity. Further, we found that higher serum klotho levels were associated with measures of greater intrinsic connectivity in key functional networks of the brain vulnerable to aging and AD such as the fronto-parietal and default mode networks. Our findings suggest that elevated klotho promotes a resilient brain, possibly through increased network connectivity of critical brain regions.

**Electronic supplementary material:**

The online version of this article (doi:10.1007/s11682-016-9598-2) contains supplementary material, which is available to authorized users.

## Introduction

Cognitive decline has emerged as a major biomedical challenge as the global population ages. However, there is striking variation in vulnerability to cognitive decline – and some individuals remain remarkably resilient to it (Jopp et al. 2016). Understanding what underlies brain resilience against aging and neurodegenerative conditions such as Alzheimer’s disease (AD) may reveal novel pathways to predict and promote cognitive health with advancing age.

The longevity factor klotho is a pleiotropic protein that suppresses aging (Kuro-o et al. 1997; Kurosu et al. 2005), enhances cognitive functions (Dubal et al. 2015; Dubal et al. 2014; Yokoyama et al. 2015), and promotes resilience against pathogenic proteins related to AD (Dubal et al. 2015; Zeldich et al. 2014). Klotho circulates throughout the brain and body (Kuro-o et al. 1997; Kurosu et al. 2005; Matsumura et al. 1998; Shiraki-Iida et al. 1998) following cleavage from its transmembrane form (Chen et al. 2007). Systemic levels of klotho decline with human aging (Semba et al. 2011; Yamazaki et al. 2010) and cognitive dysfunction (Shardell et al. 2015) and are modified by the environment (Matsubara et al. 2014; Prather et al. 2015) and genetics (Dubal et al. 2014).

A functional haplotype in human *KLOTHO* (*KL*) formed by two non-synonymous variants, rs9536314 (F352 V) and rs9527025 (C370S), alters klotho protein secretion (Arking et al. 2002; Dubal et al. 2014) and may change its functions (Arking et al. 2002; Tucker Zhou et al. 2013). Carrying one copy of the KL-VS haplotype is associated with longevity (Arking et al. 2005; Arking et al. 2002; Invidia et al. 2010), healthy cardiovascular functions (Arking et al. 2005; Arking et al. 2002), better renal health (M. C. Hu et al. 2012), and increased global cognition in healthy older adults (Dubal et al. 2014; Yokoyama et al. 2015). For unknown reasons, KL-VS homozygosity is associated with shortened lifespan, harmful health effects (Arking et al. 2005; Arking et al. 2002; Deary et al. 2005; Majumdar et al. 2010), and decreased cognitive function (Deary et al. 2005; Yokoyama et al. 2015).

The genetic influence of *KL* extends to cortical brain structures vulnerable to aging (Yokoyama et al. 2015). Carrying one copy of KL-VS is associated with greater volume in right dorsolateral prefrontal cortex (rDLPFC) along with better executive function when compared to individuals with no copies (Yokoyama et al. 2015). rDLPFC drives executive function, a type of cognition used for planning and organizing that is targeted in aging (Hu et al. 2014; Morrison and Baxter 2012; Müller-Oehring et al. 2013; Taki et al. 2013) and AD (Lunnon et al. 2014; Perry and Hodges 1999). Relatedly, this prefrontal region also influences cognitive action control (Cieslik et al. 2013), working memory (Fried et al. 2014; Hillary et al. 2006; Honey et al. 2000), motor learning (Lin et al. 2012), and emotion regulation (Lévesque et al. 2003). Interestingly, and in parallel with its link to poor health outcomes, KL-VS homozygosity is correlated with smaller rDLPFC volume and worse executive function (Yokoyama et al. 2015).

The goals of this study were to assess the relationship between systemic klotho levels and KL-VS genotype (heterozygosity and homozygosity) and to ascertain the neuroanatomical correlates of serum klotho levels in healthy cognitive aging. We assessed serum klotho levels in non-carriers, KL-VS heterozygotes, and KL-VS homozygotes. We then identified volumetric differences in cortical gray matter (GM) associated with klotho serum level and identified other regions of the brain functionally “linked” to these GM regions via measures of intrinsic connectivity. Finally, we assessed how these functional connections vary based on serum klotho levels. We found that klotho levels are increased with KL-VS heterozygosity, as anticipated (Dubal et al. 2014), and report, for the first time, that levels are paradoxically decreased with KL-VS homozygosity. Further, we found that higher serum klotho levels are associated with greater intrinsic connectivity of rDLPFC and right temporal lobe to functional networks most affected in aging.

## Materials and methods

### Participants

Healthy older adults are participants in on-going research on determinants of healthy cognitive aging at the University of California, San Francisco (UCSF) Memory and Aging Center (MAC). All participants undergo a multi-step screening as healthy older adults, with an in-person visit at the MAC that includes a neurologic exam, detailed cognitive assessment (Rankin et al. 2005), and medical history. Each participant’s study partner is interviewed regarding functional abilities. A consensus team of clinicians then reviews all potential participants. Each participant also undergoes a detailed family history for neurodegenerative disease, and is followed on an annual basis. Participants in this study were between ages 55 and 90-years-old; had a study partner (i.e., spouse, close friend); no participant or informant report of cognitive decline in the prior year; and no evidence from screening visit suggesting a neurodegenerative disorder (per team neurologist’s clinical judgment). Individuals with a family history of autosomal dominant neurodegenerative or neuropsychiatric disease and individuals harboring a known disease mutation were excluded from study. In this study, participants were White, reflecting the primary demographic of our study participants. All participants provided written informed consent to participate in research and the university’s institutional review board approved all aspects of this study.

### Genotypes

Genomic DNA was extracted from whole blood using standard methods. KL-VS genotyping of rs9536314 for F352V and rs9527025 for C370S was performed using Sequenom iPLEX Technology and manufacturer’s instructions as described (Sequenom, San Diego, CA) (Yokoyama et al. 2015).

### Serum measurements

Using published methods (Dubal et al. 2014; Prather et al. 2015), soluble α-klotho (α-klotho), the major circulating form of klotho, was measured using a solid-phase sandwich enzyme-linked immunosorbent assay (Immuno-Biological Laboratories, Takasaki, Japan) (Yamazaki et al. 2010) according to manufacturer instructions in serum from morning fasting blood samples. Serum was diluted 4-fold with the supplied Enzyme Immunoassay buffer. A standard curve was established by serial dilution of recombinant human soluble α-klotho protein. In-house standards and repeated samples were included to control for inter-plate variability. Some data obtained from serum klotho levels of non-carriers and KL-VS heterozygotes were previously reported (Dubal et al. 2014).

### Image acquisition

MR images were acquired on a 3 T Siemens Tim Trio system equipped with a 12-channel head coil at the UCSF Neuroscience Imaging Center. A volumetric MPRAGE sequence was used to acquire T1-weighted images (1 × 1 × 1 mm voxel size; FOV = 256 × 240 mm and 160 slices, TR = 2300 ms, TE = 3 ms, FA = 9°). Resting state functional MRI scans data were acquired (2. 5 × 2.5 × 3 mm voxel size; FOV = 230 × 230 mm, TE = 27 ms, TR = 2 s, FA = 80°) with 36 interleaved axial slices. Resting state scans were 8 min in duration, capturing 240 images. Participants were instructed to remain awake with their eyes closed for the duration of the scan.

### Image processing

To assess the effect of klotho serum levels on GM volume, we conducted whole-GM voxel-based morphometry (VBM) analysis using SPM12 (Ashburner and Ridgway 2013; Ashburner 2012) (http://www.fil.ion.ucl.ac.uk/spm/software/spm12/) along with the DARTEL toolbox (Ashburner 2007). T1-weighted structural images were segmented then warped to a custom healthy older adult DARTEL template using default settings. DARTEL-processed GM images were linearly coregistered to the MNI152 template and smoothed with a 6 mm FWHM kernel. Images were manually validated for accurate segmentation and registration. Modulated images were used for all analyses, where GM volume is assessed through a continuous measure (from 0 to 1) of a given voxel’s probability of being GM. Extracted volume measures are a mean of the probability of being GM across a specified region of interest (ROI) and were used to further quantify klotho effects in secondary analyses.

For resting state analysis, the first five functional images were discarded to allow for magnetic field stabilization. Functional images were slice-time corrected, spatially realigned, coregistered to the structural template, warped to MNI template space, and subsequently smoothed with a 6 mm FWHM kernel all using the SPM12 toolbox. Images then underwent a temporal band pass filter (0.008–0.15 Hz) using FSL (Jenkinson et al. 2012) (http://fsl.fmrib.ox.ac.uk/fsl). Processed images were then manually validated for accurate registration and excessive motion. Participants’ entire time series were discarded if there was over 3 mm relative head motion or motion spikes (relative motion >1 mm) occurring in over 10 % of their total frames.

### Statistical analysis

All voxel-based statistics were carried out using vlsm2.55 (Bates et al. 2003). General linear models were fit to each voxel modeling the dependence of either tissue density or seed-connectivity on klotho serum levels. VBM analysis was covaried with total intracranial volume (TIV) as well as age and sex while intrinsic connectivity map analyses were covaried with age and sex. In secondary VBM assessment, KL-VS genotype was coded to model the putative biological relationship of each group (KL-VS homozygotes < KL-VS non-carriers < KL-VS heterozygotes as 1, 2, or 3, respectively) (Yokoyama et al. 2015) and covaried for TIV, age, and sex.

For seed-based functional connectivity analysis, ROIs for seed-based analysis were selected as voxels with the strongest correlation between klotho serum and tissue density. A 4 mm radius sphere centered on the peak voxel coordinate from the structural analysis was created and used as the seed in the seed-to-voxel resting state analysis. To assess intrinsic connectivity for each seed ROI, the average time series was de-trended and used as a covariate of interest in a whole-brain statistical parametric analysis. This generated intrinsic connectivity maps for each participant, in which each voxel was scored based on the level of correlation between its time-series and the time-series of the ROI seed. Nuisance regressors were calculated for cerebrospinal fluid (CSF) using a seed located in the lateral ventricles and for the white matter using a seed in the highest probability of FSL’s standard tissue probability map. The six motion parameters calculated in the realignment process were also included as nuisance regressors. Additionally, temporal derivatives of the eight regressors (2 tissue + 6 motion) as well as the squares of those 16 terms were included as nuisance regressors in the seed connectivity analysis (Satterthwaite et al. 2013).

Summary statistics and assessment of klotho serum levels as a function of KL-VS genotype were performed in Stata10/MP (StataCorp, College Station, TX, USA) and GraphPad Prism 6 (GraphPad Software, Inc.). We corrected for the multiple comparisons of post-hoc tests with the Benjamini-Hochberg procedure using R (nmle package). Quantification of imaging results was performed on extracted measures of GM volume (for structural images) or strength of connectivity to seed region (for functional imaging) for the ROI and then used in secondary linear regression and partial correlation analyses to further quantify the strength of correlation between klotho serum level and structural/functional measures. Analyses and visualization were performed in Stata10 and GraphPad Prism.

## Results

### Klotho serum level is highest in KL-VS heterozygotes and lowest in KL-VS homozygotes

**Fig. 1:**
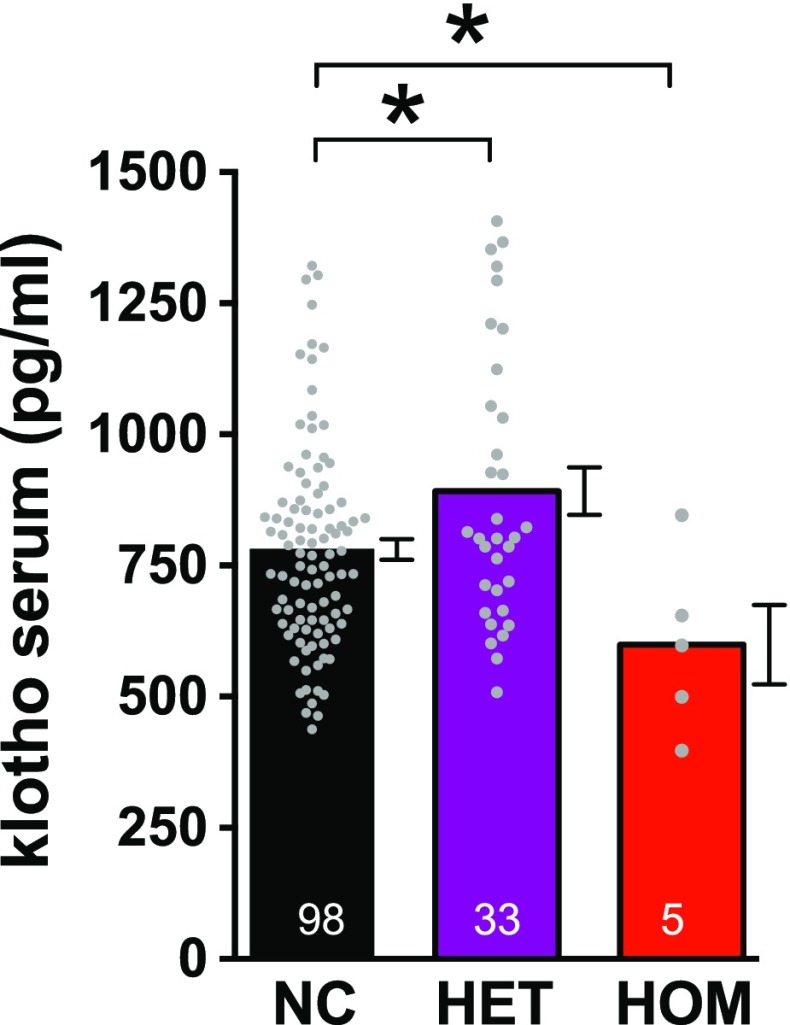
Klotho serum level is highest in KL-VS heterozygotes and lowest in KL-VS homozygotes. Serum klotho levels from fasting morning serum samples of healthy older adults include 98 KL-VS non-carriers (*black*), 33 KL-VS heterozygotes (*purple*), and 5 KL-VS homozygotes (*red*); individual values are shown as grey dots. There was a significant effect of KL-VS genotype on klotho serum levels (*p* = 0.004 unadjusted; *p* = 0.007 adjusted for age, sex, and *APOE* ε4 dose). Post hoc pair-wise comparisons with non-carriers showed that KL-VS heterozygotes had significantly elevated klotho and KL-VS homozygotes had significantly lower klotho serum levels (*p < 0.05 vs non-carriers, Benjamini Hochberg procedure). Data from individual non-carriers and heterozygotes was included in Dubal et al. (2014). Data are Mean ± SEM

In total, 136 individuals had KL-VS genotypes, klotho serum, and combined structural and functional imaging data available for analysis. On average, these individuals were 74 years old, 49 % female and highly educated (Table 1). We found that klotho serum levels varied by KL-VS genotype (*p* = 0.004, F(2133) = 5.76, two-tailed ANOVA). The effect of genotype remained significant after accounting for age at serum draw, sex, and *APOE* ε4 dose (ANOVA, KL-VS *p* = 0.007). As anticipated (Dubal et al. 2014), KL-VS heterozygotes had higher klotho serum levels than non-carriers (mean ± se 891.7 ± 45.3 pg/ml in 33 heterozygotes versus 780.9 ± 19.6 pg/ml in 98 non-carriers; Fig. 1). Additionally, we found that KL-VS homozygotes had the lowest average levels of klotho serum (599.1 ± 75.7 pg/ml in 5 homozygotes), with significantly lower levels than non-carriers (*p* < 0.05 post hoc pair-wise comparison). There was no interaction between KL-VS genotype and sex or age to predict serum level, though these analyses may be limited in power. Thus, systemic klotho measured by klotho serum level was increased with one KL-VS allele of *KL* but paradoxically decreased with two KL-VS alleles.

**Table 1:** Descriptive statistics of study participants

	Non-carriers	KL-VS heterozygotes	KL-VS homozygotes	*p*-value
N	98	33	5	
Age (mean ± se)	74.0 ± 0.7	74.2 ± 1.1	72.0 ± 4.0	0.78
Sex (n female)	53	12	2	0.19
Edu (years)	17.8 ± 0.2	17.3 ± 0.4	17.2 ± 1.2	0.43
*APOE* ε4 alleles (0 / 1 / 2)	74 / 21 / 3	26 / 7 / 0	4 / 1 / 0	0.88

### Higher systemic klotho level predicts greater GM volume in multiple cortical regions

**Fig. 2:**
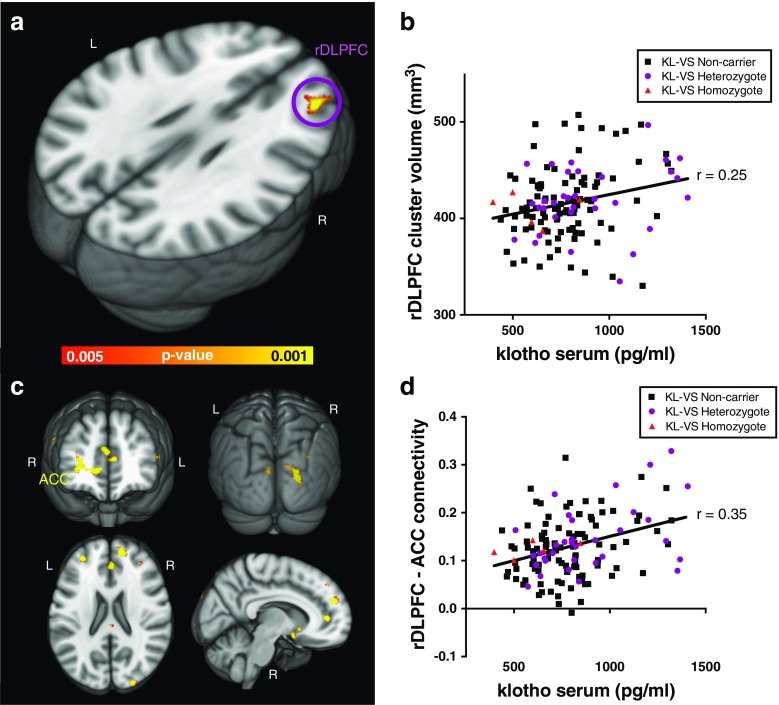
rDLPFC shows greater volume and connectivity to ACC with higher klotho serum levels**. a** Higher serum klotho levels are associated with greater volume in right dorsolateral prefrontal cortex (rDLPFC), highlighted in purple circle and shown as a red-yellow heat map representing *p*-value of association. **b** Klotho serum level is positively correlated with rDLPFC volume across all genotypes (Pearson’s correlation coefficient *r* = 0.25). **c** Higher klotho serum levels are associated with greater intrinsic connectivity (represented by heat map of association *p*-value) between the rDLPFC seed and other regions of the fronto-parietal functional network, including the dorsal anterior cingulate cortex (ACC, yellow text). **d** Representative connectivity between rDLPFC and ACC is positively correlated with klotho serum level across genotypes (*r* = 0.35). All imaging results are overlaid on the MNI152 template

**Fig. 3:**
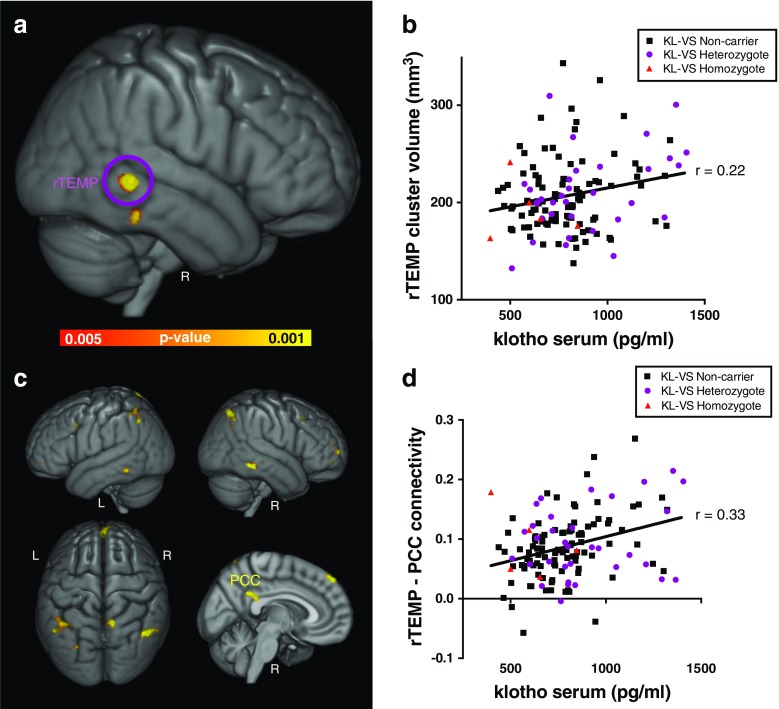
rTEMP shows greater volume and connectivity to PCC with higher klotho serum levels. **a** Higher serum klotho levels are associated with greater volume in right middle temporal gyrus (rTEMP), highlighted in purple circle and shown as a red-yellow heat map representing *p*-value of association. **b** Klotho serum level is positively correlated with rTEMP volume across all genotypes (Pearson’s correlation coefficient *r* = 0.22). **b** Higher klotho serum levels are associated with greater intrinsic connectivity (represented by heat map of association p-value) between the rTEMP seed and other regions of the default mode network, including the posterior cingulate cortex (PCC, yellow text). **d** Representative connectivity between rTEMP and PCC is positively correlated with klotho serum level across genotypes (*r* = 0.33). All imaging results are overlaid on the MNI152 template

To identify correlations between systemic klotho and GM volume, we performed voxel-wise analysis. Higher serum klotho level correlated with greater GM volume in multiple brain regions (Table 2). Peak correlations (p_uncorr_ < 0.001) were found in rDLPFC (MNI coordinates: 28, 45, 28; Fig. 2A-B), similar to previous findings with KL-VS genotype (Yokoyama et al. 2015). Higher serum klotho also predicted greater volume in right middle temporal gyrus (rTEMP; p_uncorr_ < 0.001, MNI: 66, −38, −3; Fig. 3A-B). Findings did not withstand multiple testing corrections via 1000 permutations, likely due to the limited sample size. Nevertheless, because of the functional relevance of these regions in cognitive aging we focused on these top two regions for our analysis of klotho serum as a predictor of intrinsic connectivity, using each of these regions as an ROI “seed” in separate analyses.

**Table 2:** Higher serum klotho level predicts greater gray matter volume. Shown in order of cluster size at unadjusted *p* < 0.001 (minimum cluster size >50 mm^3^). rTEMP (right temporal lobe); rDLPFC (right dorsolateral prefrontal cortex, including right middle frontal gyrus)

Volume (in mm^3^)	x	y	z	Region	Max T
263	66	-38	-3	rTEMP	4.63
118	28	45	28	rDLPFC	3.46
105	-5	-60	63	Left precuneus	3.75
57	21	60	26	rDLPFC	3.82

### Higher systemic klotho predicts greater connectivity between rDLPFC to multiple functional networks

Intrinsic connectivity of the rDLPFC seed region across the rest of the brain is shown in Fig. S1. Higher serum klotho level was significantly correlated (p_uncorr_ < 0.001) with greater rDLPFC connectivity to medial frontal areas of the brain, with peaks in anterior cingulate cortex (ACC; Max T-score: 4.52; MNI: 2, 38, 24) and right middle frontal gyrus (Max T-score: 4.15; MNI: 30, 40, 14; Fig. 2C-D). rDLPFC connectivity to the occipital lobe was also significantly enhanced (Max T-score: 3.72; MNI: 26, −92, 22). These findings all occurred within the rDLPFC seed network (Fig. S3 A). They did not withstand multiple testing corrections. Accounting for volume of the rDLPFC seed region in the analysis did not alter the results (data not shown).

### Higher systemic klotho level predicts greater connectivity between right temporal lobe and the functional network affected in Alzheimer’s disease

Intrinsic connectivity of the rTEMP seed region across the rest of the brain is shown in Fig. S2. In the same group of individuals, higher serum klotho was also associated with greater intrinsic connectivity between the rTEMP seed (p_uncorr_ < 0.001; Fig. 3C-D) and posterior cingulate cortex (PCC; Max T-score: 3.82; MNI: 4, −40, 28), as well as medial portions of superior frontal cortex (Max T-score: 4.11; MNI: 4, 50, 44), left middle temporal gyrus (Max T-score: 3.90; MNI: −36, −70, 26), and bilateral inferior parietal lobules (Left: Max T-score: 3.81; MNI: −48, −60, 42; Right: Max T-score: Right: 3.01, p_uncorr_ < 0.005; MNI: 56, −60, 32). All of these findings occurred within the rTEMP seed network (Fig. S3B). Together, these regions constitute key nodes of the default mode network (DMN), the functional group of neuroanatomical regions engaged during internally focused tasks and affected in AD (Buckner et al. 2008; Greicius et al. 2004). These findings did not withstand correction for multiple testing through permutation likely due to limited sample size. Accounting for volume of the rTEMP seed region did not alter these results (data not shown). These findings suggest that elevated systemic klotho promotes a resilient brain, possibly through increased network connectivity.

## Discussion

Our data show that systemic klotho levels, measured in serum, are increased with KL-VS heterozygosity, as reported (Dubal et al. 2014), and indicate, for the first time, that levels are paradoxically decreased with KL-VS homozygosity. Further, we found that higher serum klotho is associated with enhanced intrinsic connectivity to rDLPFC and rTEMP in healthy aging. Together, our results suggest that elevated klotho increases brain resilience to aging or AD, at least in part, by enhanced size and network connectivity of the prefrontal and temporal cortex.

KL-VS heterozygosity is associated with increased systemic klotho and confers advantage in brain health. Indeed, studies in mice show that elevating wildtype klotho causes cognitive enhancement (Dubal et al. 2015; Dubal et al. 2014) and synaptic resilience against pathogenic proteins related to AD (Dubal et al. 2015). It is also possible that KL-VS via two amino acid substitutions can change klotho function (Zhou et al. 2013), a possibility that remains to be tested in animal models. It is known that elevated wildtype klotho exerts a myriad of biologic functions including suppression of insulin signaling (Kurosu et al. 2005), activation of growth factor signaling (Kurosu et al. 2006), enrichment of synaptic NMDA receptor subunits (Dubal et al. 2015; Dubal et al. 2014), and trafficking of other ion channels (Chang et al. 2005; Imura et al. 2007). How these klotho-mediated biologic functions relate to each other and to advantages in brain health are important lines of investigation.

Our finding that lower serum klotho is associated with KL-VS homozygosity is consistent with the shortened lifespan (Arking et al. 2002), increased disease risk (Deary et al. 2005; Invidia et al. 2010; Majumdar et al. 2010), and decreased cognition (Deary et al. 2005; Yokoyama et al. 2015) associated with this genotype. Reasons for the paradoxical dose effect of carrying two KL-VS alleles on klotho levels are not known. Possibilities include diminished ability to secrete cellular klotho (Arking et al. 2002) or decreased overall klotho production due to variant homozygosity (but increased compensatory production of the wildtype *KL* allele with variant heterozygosity).

Neuroimaging analysis revealed that higher klotho serum level predicts greater GM volume in rDLPFC and in rTEMP in aging. These findings, in part, extend previous studies showing that KL-VS heterozygosity is associated with greater rDLPFC volume along with other cortical regions (Yokoyama et al. 2015). rDLPFC is well established as a vulnerable region in aging (McEwen and Morrison 2013; Morrison and Baxter 2012; Young et al. 2014), and is functionally linked to multiple brain networks that play diverse roles in cognition related to decision-making, cognitive and motor coordination, and emotional regulation. The specific rDLPFC location reported in this study is most likely situated within the fronto-parietal network, which supports cognitive control and decision-making (Dosenbach et al. 2007; Vincent et al. 2008; Yeo et al. 2011). The other region associated with klotho levels, rTEMP, is a node in the DMN, which activates during internally-oriented tasks such as autobiographical memory retrieval, envisioning the future, and taking the perspectives of others (Buckner et al. 2008). Collectively, rDLPFC and rTEMP loci span systems related to both internally- and externally-oriented cognition, indicating diversity of klotho-associated networks.

When rDLPFC and rTEMP were probed in separate analyses, we found that higher klotho was associated with greater intrinsic connectivity involving each region. Higher klotho predicted enhanced connectivity between rDLPFC and multiple functional networks. DLPFC is a multimodal association hub region, well positioned to participate in and integrate information from diverse functional systems (Buckner et al. 2009; Power et al. 2011). Here rDLPFC’s connectivity was particularly elevated to the dorsal ACC, another component of the fronto-parietal network. Impaired cognitive control in aging and neurodegenerative disease occurs via abnormal prefrontal functioning and affects a wide range of cognitive processes including working memory, inhibition, speed of processing, and attention (Braver and Barch 2002; Grady 2012). It is possible that klotho-driven enhancement of fronto-parietal network connectivity enables greater cognitive control.

Higher klotho levels also predicted enhanced connectivity between rTEMP and the DMN, regions that are selectively vulnerable to degeneration in AD (Seeley 2008; Seeley et al. 2009). Changes in DMN activity are relevant to multiple disorders including depression, autism, schizophrenia, and attention-deficit/hyperactivity disorder; further, the DMN is the primary network of brain regions affected by neurodegeneration in AD (Buckner et al. 2008). In AD, the DMN demonstrates increased amyloid deposition, reduced glucose metabolism, and reduced functional connectivity (Buckner et al. 2005; Greicius et al. 2004; Sheline et al. 2010). It is interesting to speculate that enhanced connectivity between rTEMP and the DMN with higher klotho levels confers neural resilience against clinical symptoms of AD – a possibility that remains to be tested.

This study utilized a well-characterized cohort of cognitively robust healthy aging individuals with multiple biomarkers and neuroimaging measures (Duarte et al. 2006; Kramer et al. 2007; Mirsky et al. 2011; Song et al. 2015; Yokoyama et al. 2014). While we speculate that our findings would extrapolate widely, caveats include inclusion of primarily Whites within northern California in the United States, raising the possibility that more diverse genetic and environmental influences could mask effects in other populations. Another caveat is the low frequency of KL-VS homozygotes in the population (~5 %), which parallels previous reports of low frequency of this genotype (Dubal et al. 2014; Freathy et al. 2006; Invidia et al. 2010; Yokoyama et al. 2015); thus, larger cohorts will be needed for well-powered studies probing effects of KL-VS homozygosity. Finally, we chose an unbiased approach to study whole-brain connectivity patterns rather than limit our focus to specific, pre-determined functional networks that have been largely established in young adults. This approach allowed us to probe for connectivity across brain regions and between putative functional networks that may have been undetected in targeted analyses. Limitations of our approach include loss of statistical power and additional interpretation required when assessing results. This loss of power resulted in our neuroimaging findings not maintaining significance after multiple testing corrections through permutation of the full linear model. Nevertheless, given the importance of the implicated neuroanatomical regions in cognitive aging and their relationship to previous work, we believe these findings provide strong initial support for a role of systemic klotho in healthy brain aging and will benefit from further study.

Though KL-VS genotype and klotho serum levels showed similar associations with neuroanatomy in healthy aging, we found that klotho levels were more robust, and independent, predictors of brain volume when compared to *KL* genotype. This highlights the variability observed across genotype groups and suggests that klotho serum level may have a closer biological relationship with brain structure and function. Interestingly, klotho serum levels and KL-VS genotype were independently associated with the phenotypes we examined. That is, higher levels of systemic klotho were associated with greater brain volume and connectivity regardless of KL-VS genotype. Similarly, the effect of KL-VS genotype persisted after accounting for serum klotho levels. Among many possibilities, this suggests that genotype may contribute developmental, organizing effects while serum levels (which are modifiable by environmental factors) (Matsubara et al. 2014; Prather et al. 2015) may exert acute, activational, and potentially reversible effects. These intriguing possibilities remain to be tested. Taken together, these findings support complementary but independent roles of KL-VS genotype and klotho serum level on healthy brain aging.

In summary, our data further support the role of elevated systemic klotho in healthy brain aging. Our findings suggest that elevated klotho promotes a resilient brain, possibly through increased network connectivity of brain regions vulnerable in aging and AD. Understanding how to elevate systemic klotho or boost its functions may represent new paths to increasing resilience in aging and diseases of aging such as Alzheimer’s.

## Electronic supplementary material

ESM 1 Figure S1 (DOCX 1353 kb)

ESM 2 Figure S2 (DOCX 1329 kb)

ESM 3 Figure S3 (DOCX 5019 kb)
