# Supplementary material for: Systemic klotho is associated with KLOTHO variation and predicts intrinsic cortical connectivity in healthy human aging
Source: Brain Imaging Behav. 2016 Oct 6;11(2):391–400. doi: 10.1007/s11682-016-9598-2 (PMC5382127; doi:10.1007/s11682-016-9598-2)
Supplement: Supplementary file 1 — (DOCX 1353 kb) [file 11682_2016_9598_MOESM1_ESM.docx]

**SUPPLEMENTAL MATERIAL**

**Figure S1: Intrinsic connectivity between rDLPFC seed and the whole brain.**


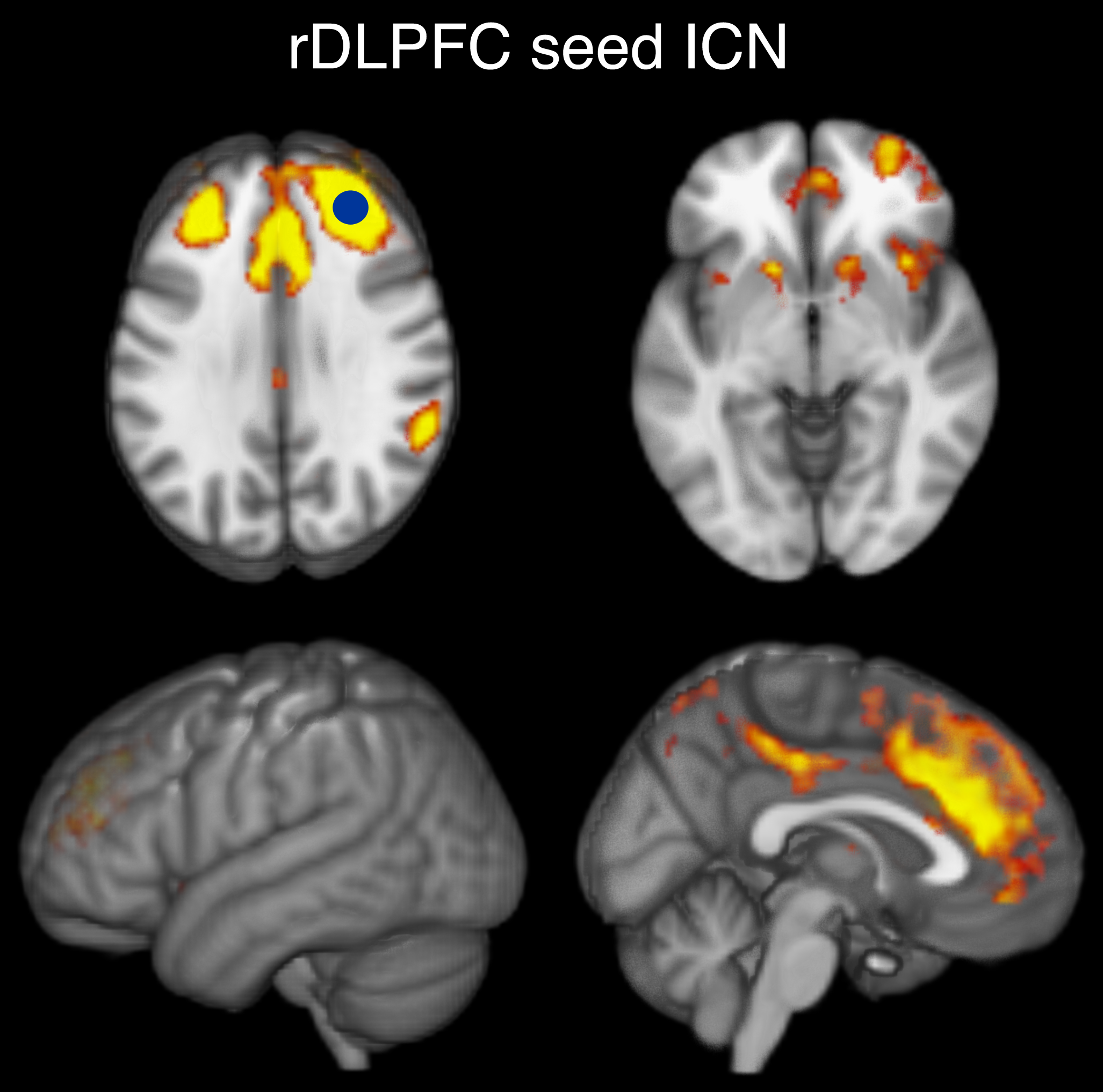


**Figure S1 Legend:** Intrinsic functional connectivity network (ICN) map anchored by right dorsolateral prefrontal cortex (rDLPFC) seed (shown as blue circle). Heat map represents regions correlated with seed for t = 15-20 (red-yellow) via one-sample t-test.
