# Supplementary material for: Systemic klotho is associated with KLOTHO variation and predicts intrinsic cortical connectivity in healthy human aging
Source: Brain Imaging Behav. 2016 Oct 6;11(2):391–400. doi: 10.1007/s11682-016-9598-2 (PMC5382127; doi:10.1007/s11682-016-9598-2)
Supplement: Supplementary file 3 — (DOCX 5019 kb) [file 11682_2016_9598_MOESM3_ESM.docx]

**Figure S3: Serum klotho-associated enhancements in intrinsic connectivity occur within relevant seed-based networks.**

**Figure S3 Legend:** Heat map of regions showing higher intrinsic connectivity with higher serum klotho levels (shown in blue, p<0.005) are overlaid on the corresponding intrinsic functional connectivity network (ICN) map anchored by (A) right dorsolateral prefrontal cortex (rDLPFC) seed or (B) right temporal lobe (rTEMP) seed (shown in red-yellow heat map). Heat map represents regions correlated with seed for t = 15-20 (red-yellow) via one-sample t-test.
